# Growth and remodeling control shape memory in morphogenetic rods

**Published:** 2026-07-27

**Authors:** Nicolas Romeo, David B. Brückner, Noah P. Mitchell

**Affiliations:** 1 Center for Living Systems, University of Chicago, IL, USA; 2 Department of Physics, University of Chicago, IL, USA; 3 Department of Molecular Genetics and Cell Biology, University of Chicago, Chicago, IL, USA; 4 NSF-Simons National Institute for Theory and Mathematics in Biology, Chicago, IL, USA; 5 Biozentrum, University of Basel, Switzerland; 6 Department of Physics, University of Basel, Switzerland; 7 Institute for Biophysical Dynamics, University of Chicago, IL, USA

## Abstract

Mechanical instabilities provide a general design principle for shaping developing organs and engineering soft materials. However, in slender structures, simple elastic buckling tends to erase rather than preserve shape complexity: structures relax to the simplest possible shape, erasing finer detail. Living systems nonetheless build complex, reproducible morphologies from continually remodeling material, while remaining robust to noise arising across scales. Using analytical theory and numerical simulations of a minimal model of growing visco-elasto-plastic rods, we show that remodeling plays two opposing roles: At low plasticity, patterns coarsen through elastic relaxation, while high plasticity converts fluctuations into geometric disorder. This sets a trade-off between shape complexity and reproducibility with an optimal intermediate plasticity, which protects initial patterns. Growth breaks this trade-off by suppressing both failure modes, enabling complex shapes to be reproducibly generated. Our results identify remodeling and growth rates as two knobs governing whether an encoded pattern is remembered, degraded, or transformed.

Living and non-living materials grow, bend, and remodel their structure to establish their shape, as seen in organs such as the gut [1–3], plants [4–6], inflatable or swelling structures [7, 8], and flexible electronics [9] alike. Both natural and engineered systems exploit the nonlinear dynamics induced by their slender geometry [6, 10–16] to obtain complex morphological outcomes, often operating in the presence of fluctuations [17–20]. When growth is slow, morphogenesis proceeds quasi-statically and geometric effects dominate [21–24]. However, soft and living systems can remodel (or flow) on the same timescale as they grow, inducing dynamical effects that can substantially alter morphologies [25–30].

Morphological dynamics are especially important in biological development, which needs to navigate a fundamental tension between growth and noise. Morphogenesis emerges from the self-organization of cells working off patterned instructions [31–33], but is executed under intrinsically stochastic conditions [34, 35]. In particular, freed from detailed balance, biological systems can tune their rheology and growth patterns to enable robust morphogenesis [36–38]. This raises a central question: How do geometric nonlinearities, rheology, and growth contribute to enable reproducible morphology starting from an initial patterned shape?

Concrete examples of “morphodynamic memory” arise across *Drosophila* morphogenesis. During germ band extension, friction between embryo and eggshell counteracts an intrinsic chiral bias [39], while tension buffering suppresses fluctuation-induced buckling [40]. In the embryonic midgut [41], early constrictions and left–right asymmetries provide spatially patterned mechanical inputs whose transformation into stereotyped loops depends on subsequent growth, confinement, and tissue remodeling [42]. Understanding the (de)stabilizing effects of properties such as growth and remodeling could help control morphogenesis of living and non-living systems alike (Fig. 1a).

To make progress, we study a minimal *plastica* model of visco-plastic or ‘morphodynamic’ rods [43], which couples a one dimensional (1D) elastica to remodeling dynamics. Internal viscosity renders the dynamics overdamped, while plastic remodeling — called viscoelasticity in mechanics [44] — lets the ‘rest’ stress-free shape adapt to the current configuration at a rate n, slowly forgetting its original form. We use this model as an analytically tractable testbed to study geometric information transmission: we prescribe an initial shape and ask how faithfully it is remembered during simulated development.

Our main finding is that remodeling is double-edged. In the absence of plasticity or growth, complex shapes relax to the fundamental buckling mode, a process that we refer to as *elastic coarsening.* Modest plasticity suppresses this effect and thereby protects complex shapes; but past a second threshold, plasticity degrades shape memory by converting noise into geometric disorder. Growth suppresses both coarsening and geometric disorder, and in doing so allows complexity and reproducibility to coexist.

## Plastica.

**FIG. 1.**
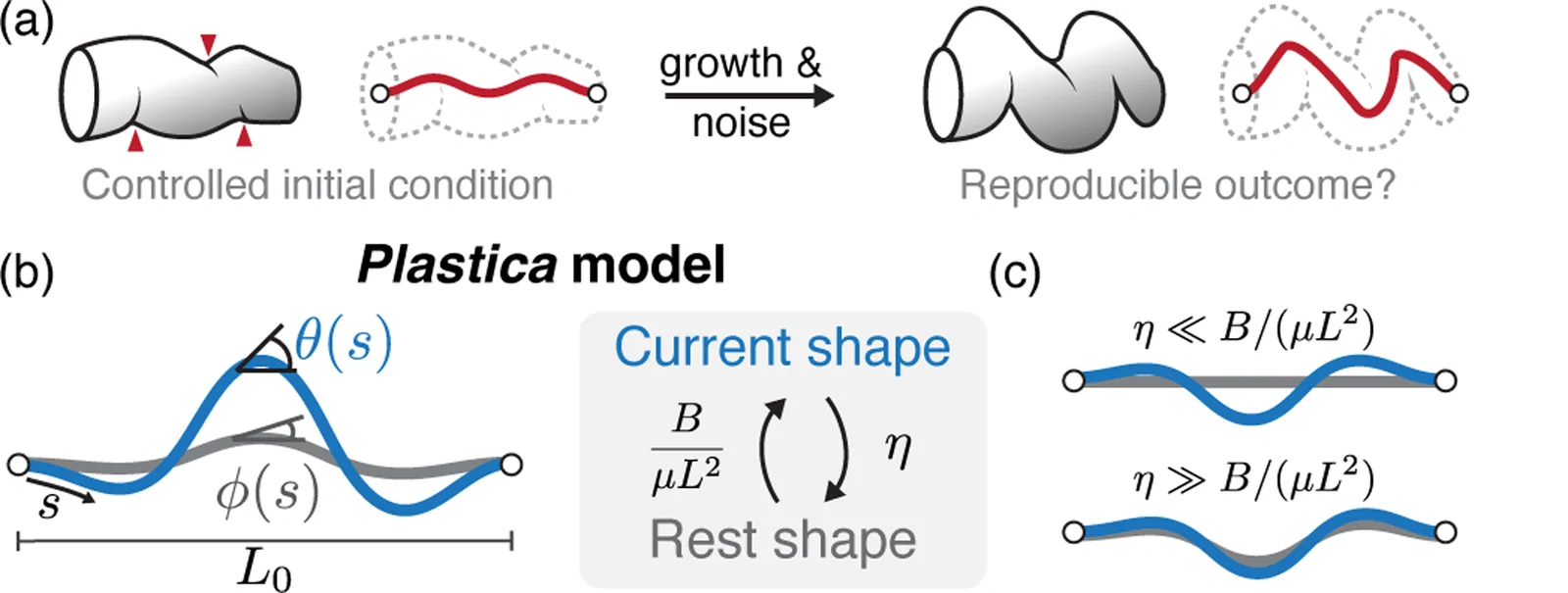
Does complex rheology stabilize noisy morphogenesis? (a) Consider an initially patterned growing 1D system. What conditions lead to reproducibly patterned outcomes? (b) We consider a minimal model of a growing elastica with internal remodeling rate n. The characteristic elastic rate is $B/(\mu L^{2})$. (c) At low plasticity $\eta \ll B/(\mu L^{2})$, the system behaves elastically, while at large η the system is fluid-like.

Inspired by computational models of viscous thread mechanics [45, 46], we consider a viscoelastic variant of the elastica: the tangent angle θ(s,t) is subject to overdamped internal viscosity μ, and the bending moment $M=B\left(\theta^{\prime }-\phi^{\prime }\right)$ is proportional to the deviation from the rest curvature $\phi^{\prime }\equiv \partial_{s}\phi$ where s is the arclength parameter (Fig. 1b). Under an imposed deformation, the rest curvature relaxes linearly to the current configuration at a rate η, causing the bending moment to vanish at long times. Varying η, the *plastica* interpolates between fully elastic (η=0) and viscous-thread (η=∞) behavior [46–48]. We consider a rod of length L with pinned (simply-supported) ends spaced apart by $L_{0}<L$. Under a small-angle approximation where $\text{Δ}\equiv L-L_{0}\ll L_{0}$, the plastica obeys

(1a) $$\mu \dot{\theta }=B\left(\theta^{\prime \prime }-\phi^{\prime \prime }\right)+F\theta +\sqrt{2\sigma }\zeta (s,t)$$

(1b) $${\dot{\phi }}^{\prime }=\eta \left(\theta^{\prime }-\phi^{\prime }\right)$$

subject to the reduced end-shortening constraint $(1/2)\int_{0}^{L}\text{d}s\;\theta^{2}=L-L_{0}\equiv \text{Δ}$, with the shorthand $\dot{\theta }\equiv \partial_{t}\theta$ and standard Brownian noise 〈ζ(s,t)〉=0, $\langle \zeta \left(s_{1},t_{1}\right)\zeta \left(s_{2},t_{2}\right)\rangle =\delta \left(s_{1}-s_{2}\right)\delta \left(t_{1}-t_{2}\right)$. While Eq. (1) appears linear, the tension F is a Lagrange multiplier imposing the quadratic constraint, making the problem nonlinear. We derive Eq. (1) from the fully nonlinear formulation valid for all $\text{Δ}/L_{0}$ in [49]; these agree in the $\text{Δ}<0.1L_{0}$ regime that follows.

The Gaussian noise here is a minimal stand-in for dynamical perturbations of the surrounding of the rod or its internal mechanics, which could be due to thermal noise or, for living systems, stochastic contractility or gene expression of proteins that set the mechanics of the system, such as filaments or motors at moderate copy counts [41, 50].

To obtain a compact description of the dynamics, we expand the tangent profile in Fourier modes $\theta (s,t)=\sum_{n=1}^{d}\theta_{n}(t)\cos\left(q_{n}s\right)$ with $q_{n}=\pi n/L$ and d the total number of modes. For 3D materials, d is set by the aspect ratio of the rod, $d\sim L_{0}/h$, with the precise form depending on the microscopic structure of the rod: thinner rods can support more modes. In mode space the dynamics become [49]

(2a) $$\mu {\dot{\theta }}_{n}=\left(-Bq_{n}^{2}+F\right)\theta_{n}+Bq_{n}^{2}\phi_{n}+\sqrt{2\sigma /L}\zeta_{n}(t)$$

(2b) $${\dot{\phi }}_{n}=\eta \left(\theta_{n}-\phi_{n}\right)$$

with the end-extension constraint now reading $\sum_{n=1}^{d}\theta_{n}^{2}=4\text{Δ}/L\equiv C$ and $\langle \zeta_{n}(t)\zeta_{m}\left(t^{\prime }\right)\rangle =\delta_{nm}\delta \left(t-t^{\prime }\right)$. The noise amplitude is length dependent in mode space: modes of a longer rod are less affected by noise as their dynamics are averaged over more microscopic kicks.

The behavior of solutions of Eq. (2) depends on a handful of dimensionless numbers, but two matter most in what follows. Defining the elastic timescale $\tau_{E}=\mu L_{0}^{2}/B$, the plasticity number $\text{Pl}=\eta \tau_{E}$ sets how fast the rod remodels relative to the speed of elastic relaxation (Fig. 1c), while a normalized growth rate $g\tau_{E}$ (introduced below) sets the speed of elongation relative to elasticity. The remaining groups – including the confinement ratio $\text{Δ}/L_{0}$, the number of modes d, and a noise scale $\bar{\sigma }=\sigma /\left(L_{0}\tau_{E}\right)$ – are held fixed unless noted. Our goal is to understand how plasticity and growth govern the fate of an initial pattern in the presence of noise.

## Elastic coarsening.

**FIG. 2.**
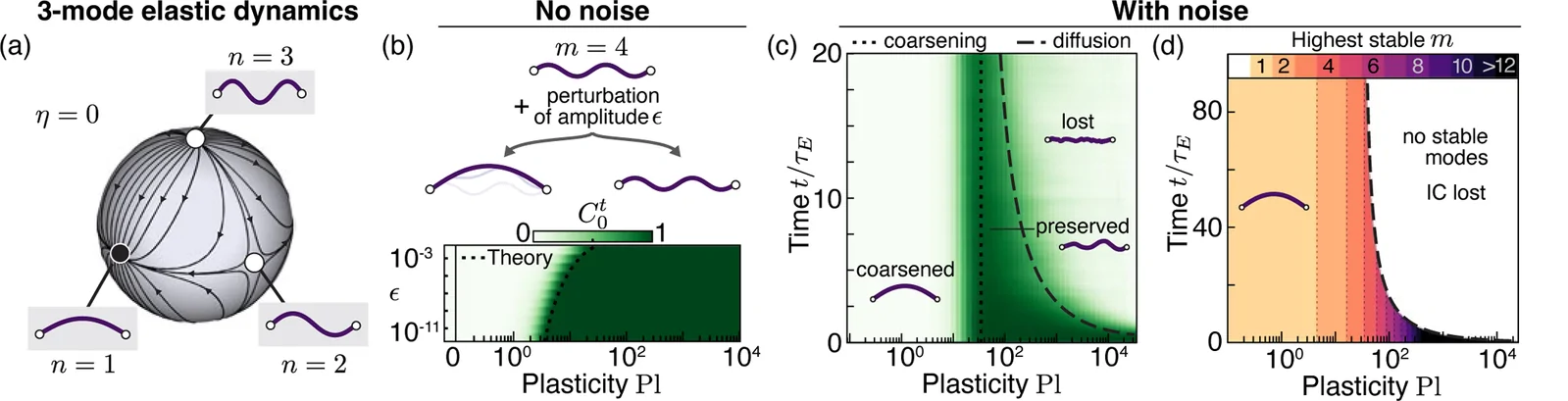
Plasticity suppresses deterministic coarsening but induces noise-driven state diffusion. (a) In the absence of growth, weakly-nonlinear dynamics of a fully-elastic system are constrained to a hypersphere, with one stable mode (black circle). Here, d=3. (b) In the absence of noise, a pure mode m>1 is robust to perturbations of amplitude ϵ only above a plasticity threshold Pl(ϵ). Here m=4. (c) High plasticity systems are vulnerable to stochastic diffusion, leading to a plasticity dependent memory time span. Boundaries derived from theory. (d) Our theory predicts that retention of the initial condition depends on its complexity, as measured by the order m of its highest mode. For readability we crop the colorbar to m=12 below its maximal value d. For (b-d), simulations averaged over 96 replicates, d=64, $L/L_{0}=1.1$ For (c-d), $\bar{\sigma }=0.005$.

We first investigate shape dynamics in the purely elastic limit η=0 with a straight rest configuration ϕ=0. The constraint $\sum_{n=1}^{d}\theta_{n}^{2}=C$ confines the mode dynamics to the sphere $S^{d-1}$. Recast in terms of the mode fractions $r_{n}=\theta_{n}^{2}/C$, the deterministic dynamics are replicator-like $\mu {\dot{r}}_{n}=2B\left({\langle q^{2}\rangle }_{r}-q_{n}^{2}\right)r_{n}$ with ${\langle q^{2}\rangle }_{r}=\sum_{n}q_{n}^{2}r_{n}$ the mode occupancy-averaged wavenumber [49]. Equation (2) has two stable fixed points, $\theta_{1}=\pm \sqrt{C}$: the fundamental mode. Every higher pure mode $\theta_{n}=\pm \sqrt{C}$ is a saddle with n−1 unstable directions flowing towards the lower modes (Fig. 2a). In analogy with population genetics, each mode has a “fitness” $f_{n}=-q_{n}^{2}$, and the smoothest, fittest mode competitively excludes the rest [51]. Any perturbation from a fixed point will therefore flow deterministically to the fundamental mode, causing elastic coarsening. Thus, complex shapes cannot be retained in the absence of plasticity.

## Plasticity protects initial patterns.

Turning on plasticity η>0 reshapes this landscape. Every configuration with $\phi_{n}=\theta_{n}$ is neutrally stable, so the entire constraint sphere $\sum_{n}\theta_{n}^{2}=C$ becomes marginally stable: once the rest shape catches up to the current configuration, the system is frozen in the absence of drive or noise, as every configuration is equivalent. The consequences are intuitive: if plasticity is fast enough so that the system reaches $\phi_{n}=\theta_{n}$ before elastic coarsening completes, then the initial condition is protected.

We make this quantitative for a rod initially in pure mode m ($\theta_{m}^{2}=C$, $\theta_{n\neq m}=0$, and $\phi_{n}=0$ for all n), subjected to an instantaneous random perturbation of typical amplitude ϵ≪1 in all modes. Plastic adaptation gives $\phi_{n}\approx \left(1-e^{-\eta t}\right)\theta_{n}$ (assuming $\theta_{n}$ changes little over this time) so the tension is $F\approx Bq_{m}^{2}\left(1-\phi_{m}/\theta_{m}\right)$ and the mode-fraction dynamics become [49]

(3) $$\mu {\dot{r}}_{n}=2B\left(q_{m}^{2}-q_{n}^{2}\right)e^{-\eta t}r_{n}.$$

Mode n stops growing after a few 1/η as $e^{-\eta t}\to 0$. For coarsening to occur before plasticity freezes the dynamics, the mode fraction $r_{1}$ must grow from its initial value $\epsilon^{2}$ to ~ 1 over this time, which translates into the condition

(4) $$\ln\left(r_{1}/\epsilon^{2}\right)=\int_{0}^{\infty }\text{d}t\frac{2B}{\mu }e^{-\eta t}\left(q_{m}^{2}-q_{1}^{2}\right)\leq \ln\left(1/\epsilon^{2}\right)$$

to prevent coarsening. Eq. (4) thus yields an m-dependent shape preservation criterion

(5) $$\frac{\eta \mu L_{0}^{2}}{B}=\text{Pl}\geq \pi^{2}{\left(L_{0}/L\right)}^{2}\left(m^{2}-1\right)/\ln(1/\epsilon ),$$

implying that more complex shapes, composed of modes with higher m, require higher plasticity to be remembered. Simulations confirm this threshold, using the Pearson correlation $C_{0}^{t}$ between initial and final modes $C_{0}^{t}=\langle \rho_{r_{n}(t),r_{n}(0)}\rangle$ averaged over replicates [52] to assess shape preservation (Fig. 2b). With noise σ>0, the same argument holds with $\epsilon^{2}$ now set by the amplitude of fluctuations $\epsilon_{\sigma }^{2}=\sigma /\left(\mu Bq_{1}^{2}C\right)$. This again matches simulations for which the threshold plasticity Pl≈30 at $\bar{\sigma }=0.005$ (Fig. 2c). Together, this shows that initial shapes are preserved beyond a minimum plasticity.

## Coarsening vs noise-sensitivity trade-off determines optimal plasticity.

While a minimum plasticity is required to avoid elastic coarsening, simulations reveal that at large plasticity the rod again loses its initial pattern due to increasing sensitivity to noise, suggesting an optimal intermediate plasticity for memory (Fig. 2c). Physically, a very plastic rod is nearly fluid and stochastic perturbations become rapidly baked into the rest configuration; mathematically, the system now diffuses freely on the constraint sphere.

To quantify this, we work in the large η limit, justified since these stochastic effects are only relevant when Pl≳30, large enough to stabilize deterministic coarsening. In this regime, the rest state approximately tracks the current state as $\phi_{n}(t)\approx \theta_{n}(t)-\eta^{-1}{\dot{\theta }}_{n}(t)$, giving effective dynamics $\gamma_{n}{\dot{\theta }}_{n}=F\theta_{n}+\sqrt{2\sigma /L}\;\zeta_{n}(t)$ [49]; with a mode-dependent ‘viscosity’ $\gamma_{n}=\mu +Bq_{n}^{2}/\eta$. Without growth, the spherical constraint forces F=0, and the system performs an anisotropic Brownian motion on the sphere, diffusing away from the initial configuration with diffusion constant $D_{d}$.

How long does it take for the system to diffuse in mode space? Correlations will be lost after a time $t_{D}∼D_{d}^{-1}$. Crucially, not all modes diffuse: high-frequency wrinkles are too stiff to be stirred by noise, and the shape diffuses in a space of lower dimension than its full mode count. Weighting each direction by its contributions to the anisotropic diffusion tensor gives $D_{d}=\left(d_{\text{eff}}-1\right)D_{0}$ with $d_{\text{eff}}(\eta )=\sum_{n=1}^{d}{\left(1+Bq_{n}^{2}/\mu \eta \right)}^{-2}$ and $D_{0}=\sigma /\left(\mu^{2}\text{Δ}\right)$. As Pl→∞, $d_{\text{eff}}\to d$ the total number of modes in the spectral representation, and diffusion becomes isotropic on the sphere; at small Pl only the fastest modes participate and $d_{\text{eff}}\to 0$. The resulting memory time $t_{D}(\text{Pl})/\tau_{E}={\left(\mu /\tau_{E}\right)}^{2}\left(\text{Δ}/L_{0}\right)/\left[\left(d_{\text{eff}}-1\right)\bar{\sigma }\right]$ agrees with simulations (Fig. 2c; see [49] for varying d and noise amplitude σ and steady-state distribution).

This memory time is independent of the initial pattern, while the threshold for protection from coarsening is m-dependent (Eq. (5)). Between the two, there is a window in which patterns are remembered, narrowing for more complex shapes with higher m≤d (Fig. 2d). Plasticity can thus protect or corrupt the memory of the initial shape, causing a trade-off between sensitivity to noise and robustness against coarsening, which depends on the complexity of the shape to be remembered.

## Growth breaks the trade-off.

A central feature of morphogenetic systems is their intrinsic growth, modeled here as uniform elongation of the rod. As the rod elongates, both the elastic coarsening rate $B/\left(\mu L{\left(t\right)}^{2}\right)$ and the spectral noise amplitude 2σ/L(t) decay. The former stems from the fact that a longer rod at a fixed mode amplitude is less curved, while the latter reflects that noise is averaged over more material. Thus, growth affects both failure modes of plasticity-based shape memory at once.

First, we consider the effect on elastic coarsening. For concreteness, we consider exponential growth $L(t)=L_{1}e^{gt}$ at a rate g from $L_{1}>L_{0}$ to a final time $T_{g}=\ln\left(L_{f}/L_{1}\right)/g$; other growth laws follow similarly. Evaluating the amplification integral Eq. (4) over the time interval $\left[0,T_{g}\right]$ gives a growth-dependent coarsening threshold: strikingly, even when Pl→0, there exists a growth rate $g_{0}$ that suppresses elastic coarsening given by $g_{0}\tau_{E}=\pi^{2}{\left(L_{0}/L_{1}\right)}^{2}\left(m^{2}-1\right)\left(1-L_{1}^{2}/L_{f}^{2}\right)/\ln\left(1/\epsilon_{\sigma }\right)$ [49]. This is consistent with the known fact that rapid compression causes higher buckling modes to be observable [53]. At large plasticity, growth-induced tension opens an alternative coarsening mechanism, but it is negligible at large Pl and mostly shifts the deterministic coarsening boundary [49]. Note here that B stays constant in time: other constitutive laws in which the rod thins as it elongates would suppress coarsening even faster [22].

**FIG. 3.**
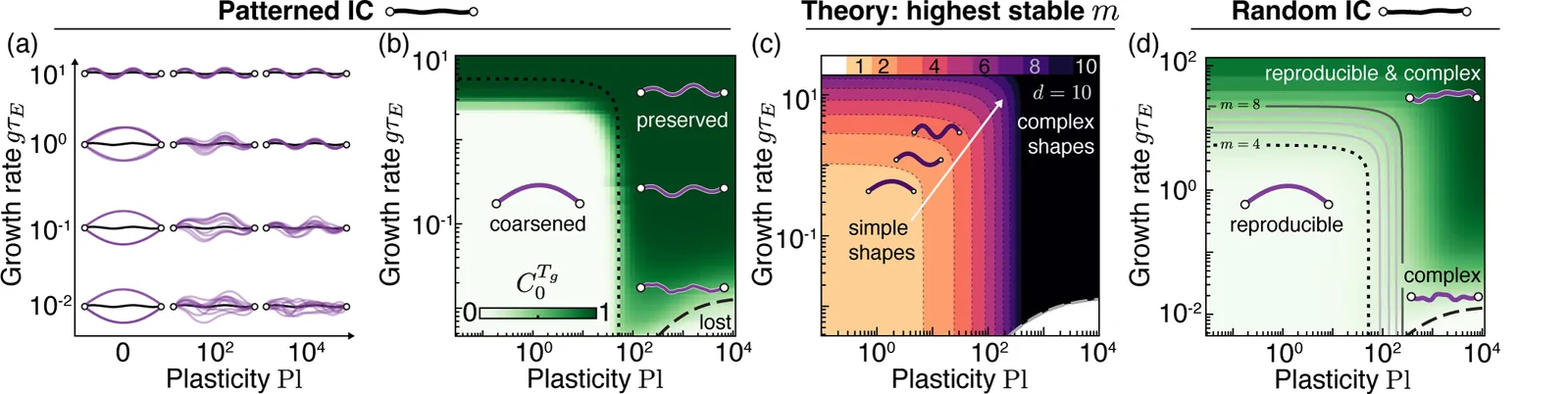
Growth stabilizes patterns. With an initial condition m=4, we find that growth and plasticity cooperate to stabilize the initial pattern. (a) Representative centerlines for 10 replicates. (b) Dotted lines indicate coarsening threshold, dashed diffusion. (c) Stabilizing increasingly complex (higher m) initial conditions (dotted and gray lines) is possible with fast growth and high plasticity. (d) Theory is consistent with numerics starting from a random initial condition, highlighting conditions for reproducible growth of complex shapes. For (a-d), $L_{1}/L_{0}=1.005$, $L_{f}/L_{1}=1.1$, $\bar{\sigma }=0.001$ and d=10. Results averaged over 96 replicates. See [49] for additional simulations with varying d and fully-nonlinear dynamics.

Second, we consider the effect on noise sensitivity. At large plasticity, noise-driven mode mixing remains dominant, and the total drift in mode space is now given by $\text{Φ}\left(T_{g}\right)=\int_{0}^{T_{g}}\text{d}t\left(d_{\text{eff}}(t)-1\right)D_{0}(t)$, which can be explicitly evaluated and must remain ≤1 for proper shape memory. In the large η limit, $d_{\text{eff}}\to d$ from below and the integral converges, giving a critical growth rate $g_{\infty }\propto (d-1)\bar{\sigma }/\tau_{E}$ above which noise can no longer erase the pattern. Explicit expressions for $g_{\infty }$ and $\text{Φ}\left(T_{g}\right)$ are provided in the supplement [49]. Both criteria are consistent with simulations starting from a single mode m=4 (Fig. 3a-b).

This analysis yields a simple design rule: to stabilize arbitrary initial conditions, there is an optimal quadrant of high growth and high plasticity. At slow growth, the plasticity trade-off is relevant: reproducible but simple shapes at low Pl, or complex but variable shapes at high Pl. Fast growth, however, enables reproducibility and complexity to go hand in hand (Fig. 3c-d).

## Discussion.

Here, we studied the influence of growth and plastic adaptation on the ability of a slender structure to preserve or transform an initially encoded pattern during development. In this formulation, the relevant question is not simply whether a tissue reaches a final shape, but whether the system retains the intended geometric program despite noise. Our minimal model gives a clear answer: remodeling protects patterns at modest rates but corrupts them above a higher threshold. Growth nullifies this trade-off between complexity and reproducibility.

This framework is relevant to systems where rheology tunes instabilities to set shape [54–56], and to the design of fluctuation-tolerant compliant materials. For example, in the *Drosophila* midgut, the results give a concrete prediction: an early constriction is amplified into a stereotyped loop only within a window of remodeling rates – fast enough that elastic relaxation does not take place, slow enough that noise is not integrated. Rapid growth should widen that window, making increasingly intricate patterns reproducible. Similar processes could be relevant for folded surfaces such as the brain’s [57, 58].

Our analysis here is restricted to a single rod with constant material properties; other dynamics can lead to more complex behavior, with external viscosity (rather than internal) already leading to intermediate coarsening [49]. Further work could explore similar effects in surfaces [27], optimal control and patterning of spatial properties [36, 59], or bundles of growing and remodeling rods [60, 61]. Finally, the mapping between buckling mode-competition and replicator dynamics [62, 63] suggests that the outcome of the interplay of elasticity, remodeling, growth, and noise found here may be relevant wherever competing components are regulated by adaptive feedback [64].

## Supplementary Material

Supplement 1
