# Supplementary material for: Growth and remodeling control shape memory in morphogenetic rods
Source: ArXiv. 2026 Jul 27:arXiv:2607.23907v1. Preprint. [Version 1] (PMC13484413)
Supplement: Supplementary file 1 [file NIHPP2607.23907v1-supplement-1.pdf]

Supplement to: Growth and remodeling control shape memory in morphogenetic rods

Nicolas Romeo,[1,2,3,4,*] David B. Brückner,[5,6] and Noah P. Mitchell[3,7,1,4,†]

[1] *Center for Living Systems, University of Chicago, IL, USA*
[2] *Department of Physics, University of Chicago, IL, USA*
[3] *Department of Molecular Genetics and Cell Biology, University of Chicago, Chicago, IL, USA*
[4] *NSF-Simons National Institute for Theory and Mathematics in Biology, Chicago, IL, USA*
[5] *Biozentrum, University of Basel, Switzerland*
[6] *Department of Physics, University of Basel, Switzerland*
[7] *Institute for Biophysical Dynamics, University of Chicago, IL, USA*

CONTENTS

I. Plastica 1
  A. Fully-nonlinear formulation 1
  B. Small-angle approximation of the plastica 2
  C. Numerical implementation 2
    1. Small-angle formulation 3
    2. Fully nonlinear formulation 3
  D. Pearson correlation 5

II. Derivation of memory criteria in the absence of growth 5
  A. Derivation of replicator equations 5
  B. Derivation of large plasticity effective dynamics 5
  C. Steady-state spectrum 7

III. Derivation of memory criterion for the growing plastica 7
  A. Elastic regime 7
  B. Large-plasticity regime with growth 8
    1. Growth in the large-plasticity regime produces limited coarsening 9
    2. State diffusion with growth 9

IV. Alternative model with external viscosity 10

References 11

I. PLASTICA

A. Fully-nonlinear formulation

We consider the elastica system, with a constitutive equation $M(s,t) = B\left(\theta'(s,t) - \phi'(s,t)\right)$, where θ is defined by the tangent vector to the curve $\mathbf{t}(s) = (\cos\theta(s), \sin\theta(s))^T$ and B is the bending modulus of the rod. Throughout, we will use dots ($\dot{\circ}$) to denote time-derivatives and primes ($\circ'$) for derivatives with respect to the curve parameter s. To model internal viscosity, we consider that the material viscosity induces an orientational relaxation timescale that dominates over the linear momentum dissipation timescale set by the viscosity of an external medium, such that the internal linear and angular momentum balance reads

$$\mathbf{F}' = 0 \tag{S1a}$$

$$M' + F_x \sin\theta - F_y \cos\theta = \mu \frac{D\theta}{Dt} \tag{S1b}$$

where D/Dt denotes the material derivative. As a consequence of the linear momentum balance Eq. (S1a), the tension of the rod $\mathbf{F} = (F_x, F_y)^\top$ is constant throughout the rod with no external viscosity. To model plasticity of the

---

* nromeo@uchicago.edu
† npmitchell@uchicago.edu

material, we append a linear update rule for ϕ such that the reference curvature ϕ' tracks the current configuration with a timescale η, which reads

$$\frac{D\phi'}{Dt} = -\eta(\phi' - \theta'). \tag{S2}$$

We consider the situation where the ends are pinned and aligned, which imposes the two constraints $\int_0^L \mathrm{d}s\, \cos\theta = L_0$ and $\int_0^L \mathrm{d}s\, \sin\theta = 0$. These constraints set the values of F_x andF_y which lead to the angle dynamics.

$$\mu\frac{D\theta}{Dt} = B(\theta'' - \phi'') + F_x \sin\theta - F_y\cos\theta \tag{S3}$$

$$\frac{D\phi'}{Dt} = \eta(\theta' - \phi') \tag{S4}$$

with $D/Dt = \partial_t + \dot{s}\partial_s = \partial_t + gs\partial_s$ for homogeneous exponential growth with $g = \dot{L}/L$. With pinned moment-free boundary conditions such that $\theta' = \phi'$ at both ends (and $\phi(0) = 0$), we can expand on cosine modes, with $q_n = \pi n/L(t)$

$$\theta(s,t) = \sum_{n=1}^{d} \theta_n(t)\cos(q_n(t)s), \quad \phi(s,t) = \sum_{n=1}^{d} \phi_n(t)\cos(q_n(t)s). \tag{S5}$$

d is the total number of modes retained in the spectral truncation, set by the physical length scale setting the validity of the one-dimensional dynamics. In mode space, we then have, accounting for the convective correction $gs\partial_s\theta$ in the presence of homogeneous growth with $g = \dot{L}/L$,

$$\mu\dot{\theta}_n = Bq_n^2(\phi_n - \theta_n) + \mathcal{F}_n[F_x\sin\theta - F_y\cos\theta] \tag{S6}$$

$$\dot{\phi}_n = \eta(\theta_n - \phi_n) \tag{S7}$$

with the inverse cosine transform $\mathcal{F}_n[f] = \frac{2}{L}\int \mathrm{d}s\, f(s)\cos(q_n s)$.

B. Small-angle approximation of the plastica

Assuming $|\theta| \ll 1$, by expanding the trigonometric functions to first order we can then reduce the equations to

$$\mu\dot{\theta}_n = (-Bq_n^2 + F)\theta_n + Bq_n^2\phi_n \tag{S8}$$

$$\dot{\phi}_n = \eta(\theta_n - \phi_n) \tag{S9}$$

with $F_y = 0$ at this order with the moment-free boundary conditions while $F = F_x$ denotes the remaining component of the tension. In the linearized regime, the inextensibility constraint simplifies to a quadratic $L_0 \approx L(t) - (1/2)\int_0^L \mathrm{d}s\, \theta^2$, and reads in terms of modes as

$$\sum_{n=1}^{d} \theta_n^2 = 4\Delta(t)/L(t) \equiv C \tag{S10}$$

with $\Delta(t) = L(t) - L_0 > 0$ the effective end-shortening. We can then find F by differentiating the constraint in time which leads to

$$F = \frac{\mu\dot{C}}{2C} + \frac{B}{C}\sum_{n=1}^{d} q_n^2\theta_n(\theta_n - \phi_n). \tag{S11}$$

C. Numerical implementation

We implement `julia` simulations to numerically solve the plastica equations, using different schemes for linear and nonlinear formulations.

1. Small-angle formulation

We use a spectral semi-implicit scheme to solve the linearized scheme: the tension is computed explicitly, but the timestepping of the ODEs is done implicitly. More precisely, at timestep k we compute $T_k = \mu\dot{C}(t_k)/C(t_k) + \frac{B}{C(t_k)}\sum_n \theta_n(t_k)(\theta_n(t_k) - \phi_n(t_k))$, then solve for the angles as

$$\mu(\theta_n^{k+1} - \theta_n^k) = (-Bq_n^2 + T_k)\theta_n^{k+1}\Delta t + Bq_n^2\phi_n^{k+1}\Delta t + \zeta_n(t_k) \tag{S12}$$

$$\phi_n^{k+1} - \phi_n^k = \eta(\theta_n^{k+1} - \phi_n^{k+1})\Delta t \tag{S13}$$

which can be recast as

$$\begin{pmatrix} \mu + (Bq_n^2 - T_k)\Delta t & -Bq_n^2\Delta t \\ -\eta\Delta t & 1 + \eta\Delta t \end{pmatrix} \begin{pmatrix} \theta_n^{k+1} \\ \phi_n^{k+1} \end{pmatrix} = \begin{pmatrix} \mu\theta_n^k + \zeta_n(t_k) \\ \phi_n^k \end{pmatrix} \tag{S14}$$

where $\zeta_n = \sqrt{2\sigma\Delta t/L}\epsilon_n$, with $\epsilon_n \sim N(0,1)$. We analytically compute the matrix inverse at each time to obtain

$$\begin{pmatrix} \theta_n^{k+1} \\ \phi_n^{k+1} \end{pmatrix} = \frac{1}{D_n}\begin{pmatrix} 1 + \eta\Delta t & Bq_n^2\Delta t \\ \eta\Delta t & \mu + (Bq_n^2 - T_k)\Delta t \end{pmatrix} \begin{pmatrix} \mu\theta_n^k + \zeta_n(t_k) \\ \phi_n^k \end{pmatrix} \tag{S15}$$

with $D_n = [\mu + (\mu\eta + Bq_n^2)\Delta t] - T_k\Delta t(1 + \eta\Delta t)$. Eq. (S15) summarizes the time stepping loop used in simulations.

2. Fully nonlinear formulation

The fully nonlinear dynamics is solved using a finite-difference approximation of the differential operators, with a nonlinear implicit Euler time stepper which requires iterative Newton solves. The arclength domain $s \in [0, L(t)]$ is sampled at N_{pts} equispaced points

$$s_i = (i-1)\,\delta, \quad i = 1, \ldots, N_{\text{pts}}, \qquad \delta = \frac{L(t)}{N_{\text{pts}} - 1}. \tag{S16}$$

The grid is regenerated every time-step as L grows and δ varies in time but the number of nodes is fixed. We use the standard second-order centered discrete Laplacian on interior points

$$(\partial_s^2\theta)_i \approx \frac{\theta_{i-1} - 2\theta_i + \theta_{i+1}}{\delta^2}, \quad i = 2, \ldots, N_{\text{pts}} - 1. \tag{S17}$$

while first derivatives at boundaries are evaluated using second-order one-sided stencils

$$(\partial_s\theta)_1 \approx \frac{-3\theta_1 + 4\theta_2 - \theta_3}{2\delta}, \qquad (\partial_s\theta)_{N_{\text{pts}}} \approx \frac{3\theta_{N_{\text{pts}}} - 4\theta_{N_{\text{pts}}-1} + \theta_{N_{\text{pts}}-2}}{2\delta}. \tag{S18}$$

These enforce the moment-free BCs $\partial_s\theta(0) = \partial_s\phi(0)$ and $\partial_s\theta(L) = \partial_s\phi(L)$ via the corresponding one-sided stencils applied to both θ and ϕ. To evaluate the constraint integral constraints, we use a composite Simpson 1/3 quadrature (if N_{pts} is odd)

$$\int_0^L f(s)\,ds \approx \frac{\delta}{3}\left[f_1 + 4f_2 + 2f_3 + 4f_4 + \cdots + 4f_{N_{\text{pts}}-1} + f_{N_{\text{pts}}}\right] \quad (N_{\text{pts}} \text{ odd}), \tag{S19}$$

with the standard pattern $(1, 4, 2, 4, 2, \ldots, 4, 1)$, falling back to trapezoidal weights when N_{pts} is even. This provides $O(\delta^4)$ accuracy on the clamp constraints for smooth integrands.

At each timestep, we analytically solve for the updated ϕ field as

$$\phi^{n+1} = \alpha\theta^{n+1} + (1-\alpha)\phi^n \tag{S20}$$

with $\alpha = \eta\Delta t/(1+\eta\Delta t)$, and then evaluate the Newton-Raphson residual for the state vector $\mathbf{u} = (\theta_1, \ldots, \theta_{N_{\text{pts}}}, F_x, F_y) \in$

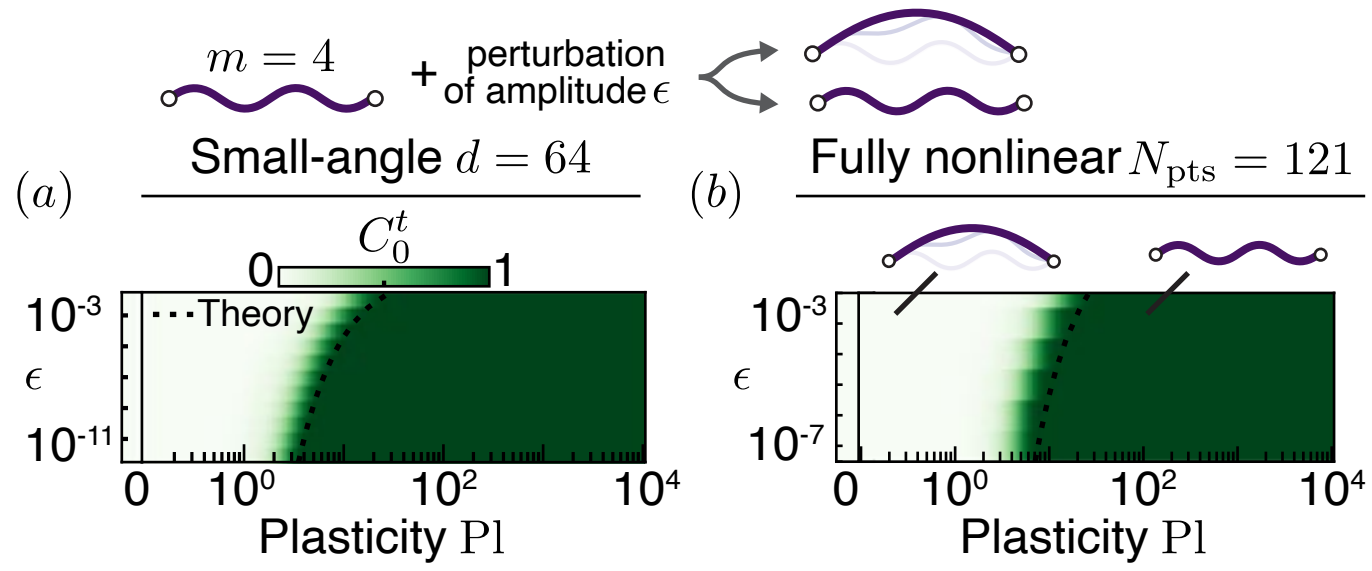

FIG. S1. Small-angle and fully nonlinear models predict similar transitions for the stability of $m = 4$ patterns in the absence of noise. For both (a) results are averaged over 96 replicates, for (b) over 36 replicates. For both, $L/L_0 = 1.1$. The range of applied perturbation amplitudes ϵ is limited in the nonlinear simulations by the accuracy of the discretization scheme.

$\mathbb{R}^{N_{\mathrm{pts}}+2}$. Defining $\bar{B} \equiv B(1-\alpha)/\delta^2$ and using (S17), the residual is

$$
\begin{aligned}
R_i^{\mathrm{interior}} &= \mu \frac{\theta_i^{n+1} - \theta_i^n}{\Delta t} - \bar{B}\left[(\theta_{i-1} - 2\theta_i + \theta_{i+1})^{n+1} - (\phi_{i-1} - 2\phi_i + \phi_{i+1})^n\right] \\
&\quad - F_x \sin\theta_i^{n+1} + F_y \cos\theta_i^{n+1}, \quad i = 2, \ldots, N_{\mathrm{pts}} - 1, && \text{(S21a)} \\
R_1^{\mathrm{BC}} &= \frac{-3\theta_1^{n+1} + 4\theta_2^{n+1} - \theta_3^{n+1}}{2\delta} - \frac{-3\phi_1^n + 4\phi_2^n - \phi_3^n}{2\delta}, && \text{(S21b)} \\
R_{N_{\mathrm{pts}}}^{\mathrm{BC}} &= \frac{3\theta_{N_{\mathrm{pts}}}^{n+1} - 4\theta_{N_{\mathrm{pts}}-1}^{n+1} + \theta_{N_{\mathrm{pts}}-2}^{n+1}}{2\delta} - \frac{3\phi_{N_{\mathrm{pts}}}^n - 4\phi_{N_{\mathrm{pts}}-1}^n + \phi_{N_{\mathrm{pts}}-2}^n}{2\delta}, && \text{(S21c)} \\
R_{F_x} &= \sum_i w_i^{\mathrm{int}} \cos\theta_i^{n+1} - L_0, && \text{(S21d)} \\
R_{F_y} &= \sum_i w_i^{\mathrm{int}} \sin\theta_i^{n+1}, && \text{(S21e)}
\end{aligned}
$$

where w_i^{int} are the Simpson weights including the arclength factor $\delta/3$. Note that since the grid expands homogeneously, the material derivative is correctly accounted by the finite difference here. The Jacobian $\mathbf{J} \in \mathbb{R}^{(N_{\mathrm{pts}}+2)\times(N_{\mathrm{pts}}+2)}$ has a banded structure with off-diagonal Lagrange-multiplier coupling, which allows us to use a sparse CSC LU solver from Julia's `SparseArrays` for the increments $\mathbf{J}(\mathbf{u}^{k+1} - \mathbf{u}^k) = -R$ where $\mathbf{u}^k$ is the k-th iteration of the Newton step.

To include noise, each time step adds a Gaussian increment to θ before the Newton solve:

$$
\theta_i^n \leftarrow \theta_i^n + \sigma_i \mathcal{N}(0,1), \qquad \sigma_i = \sqrt{\frac{2\sigma\Delta t}{V_i}}. \tag{S22}
$$

where we scale the noise amplitude to account for the variable grid spacing V_i

$$
V_i = \begin{cases} \delta & i = 2, \ldots, N_{\mathrm{pts}} - 1 \text{ (interior)} \\ \delta/2 & i = 1, N_{\mathrm{pts}} \text{ (boundary)} \end{cases}. \tag{S23}
$$

This scheme is consistent with the continuum variance rule $\langle \zeta(s,t)\zeta(s',t')\rangle = \delta(s-s')\delta(t-t')$. The Newton solve then projects the noisy θ_i^n onto the constraint manifold.

In summary, each time step $n \to n+1$ includes the following steps

1. Update $L^{n+1} = L_0 e^{g\, t^{n+1}}$, $\delta^{n+1} = L^{n+1}/(N_{\mathrm{pts}} - 1)$, and the Simpson weights w_i^{int} from (S19).
2. Add noise $\theta_i^n \leftarrow \theta_i^n + \sigma_i \mathcal{N}(0,1)$ via (S22), with the new δ^{n+1} in V_i.
3. Newton iterate (S21) until the residual $|\mathbf{R}|_\infty <$ tolerance ($\sim 10^{-8}$);
4. Recover ϕ^{n+1} from (S20).

In practice, we find that this solver is consistent with the small-angle spectral solver for perturbations of amplitude $\epsilon > 10^{-6}$ in deterministic simulations (Fig. S1). At smaller ϵ, the limited precision of this solver which estimates spatial derivatives with $O(\delta^2)$ accuracy leads to divergent results with the spectrally-accurate semi-linear small-angle solver.

D. Pearson correlation

To measure preservation of the initial condition, throughout this article we use the Pearson correlation coefficient averaged over all modes. Mathematically, we define

$$C_0^t = \langle \rho_{r_n(t),r_n(0)} \rangle \tag{S24}$$

with $\langle . \rangle$ the average over N replicates and

$$\rho_{r_n(t),r_n(0)} = \frac{1}{d} \sum_{n=1}^{d} \frac{z_n(t) z_n(0)}{\sigma_{z_n(t)} \sigma_{z_n(0)}} \tag{S25}$$

the Pearson correlation between the two mode vectors, where $z_i(t) = r_n^{(i)} - d^{-1} \sum_k r_k^{(i)}$ is the mode fraction minus the mode-averaged fraction for the i-th replicate at time t and $\sigma_{z_i(0)}$ is the per-replicate standard deviation across the mode fractions. If $r_n^{(i)}(t) = r_n^{(i)}(0)$ then $\rho_{r_n(t),r_n(0)} = 1$, while if the both arguments are uncorrelated $\langle \rho_{r_n(t),r_n(0)} \rangle = 0$. Consequently, C_0^t assesses the preservation of the initial condition at time t.

II. DERIVATION OF MEMORY CRITERIA IN THE ABSENCE OF GROWTH

In this section, we derive in more detail the deterministic and stochastic criteria for shape preservation presented in the main text. We also compare the numerically-observed steady-state spectrum in the large plasticity regime to theoretical predictions.

A. Derivation of replicator equations

We first derive the dynamics of the mode fraction $r_n(t) = \theta_n^2/C$ in the absence of growth and noise. By construction, the mode fractions satisfy $\sum_{n=1}^{d} r_n = 1$ and their dynamics thus live on the d-dimensional unit simplex. By applying the chain rule, we have

$$\mu \dot{r}_n = 2 \frac{\theta_n \dot{\theta}_n}{C} = 2 \left(F - B q_n^2 (1 - \phi_n/\theta_n) \right) r_n \tag{S26}$$

and the tension reads, per Eq. (S11) when $\dot{C} = 0$,

$$\mu \dot{r}_n = 2 \left(\sum_{k=1}^{d} B q_k^2 r_k (1 - \phi_k/\theta_k) - B q_n^2 (1 - \phi_n/\theta_n) \right) r_n. \tag{S27}$$

Assuming that plasticity is sufficiently rapid for $\phi_n = (1 - e^{-\eta t})\theta_n$ for all n, we then find the time-decaying replicator equation

$$\mu \dot{r}_n = 2B \left(\sum_{k=1}^{d} q_k^2 r_k - q_n^2 \right) e^{-\eta t} r_n. \tag{S28}$$

which reduces to standard replicator dynamics when $\eta = 0$. Here, the "species" are the bending modes, and $r_n = \theta_n^2/C$ is the fraction of the "population" in the n-th mode. Equation (S28) has the standard replicator form $\dot{r}_n = r_n \left(f_n - \bar{f} \right)$, with an effective fitness $f_n \propto -q_n^2$. Thus, long-wavelength, low-n modes increase in relative weight at the expense of shorter-wavelength modes. This redistribution is the mechanical coarsening process. When $\eta > 0$, the factor $e^{-\eta t}$ acts as a decaying selection strength, so plasticity slows and eventually arrests the redistribution.

B. Derivation of large plasticity effective dynamics

To study the interactions between plasticity and noise, we study the effective dynamics when plasticity is large enough to mostly wash out the memory of previous states. More formally, the equation $\dot{\phi}_n = \eta(\theta_n - \phi_n)$ is solved via

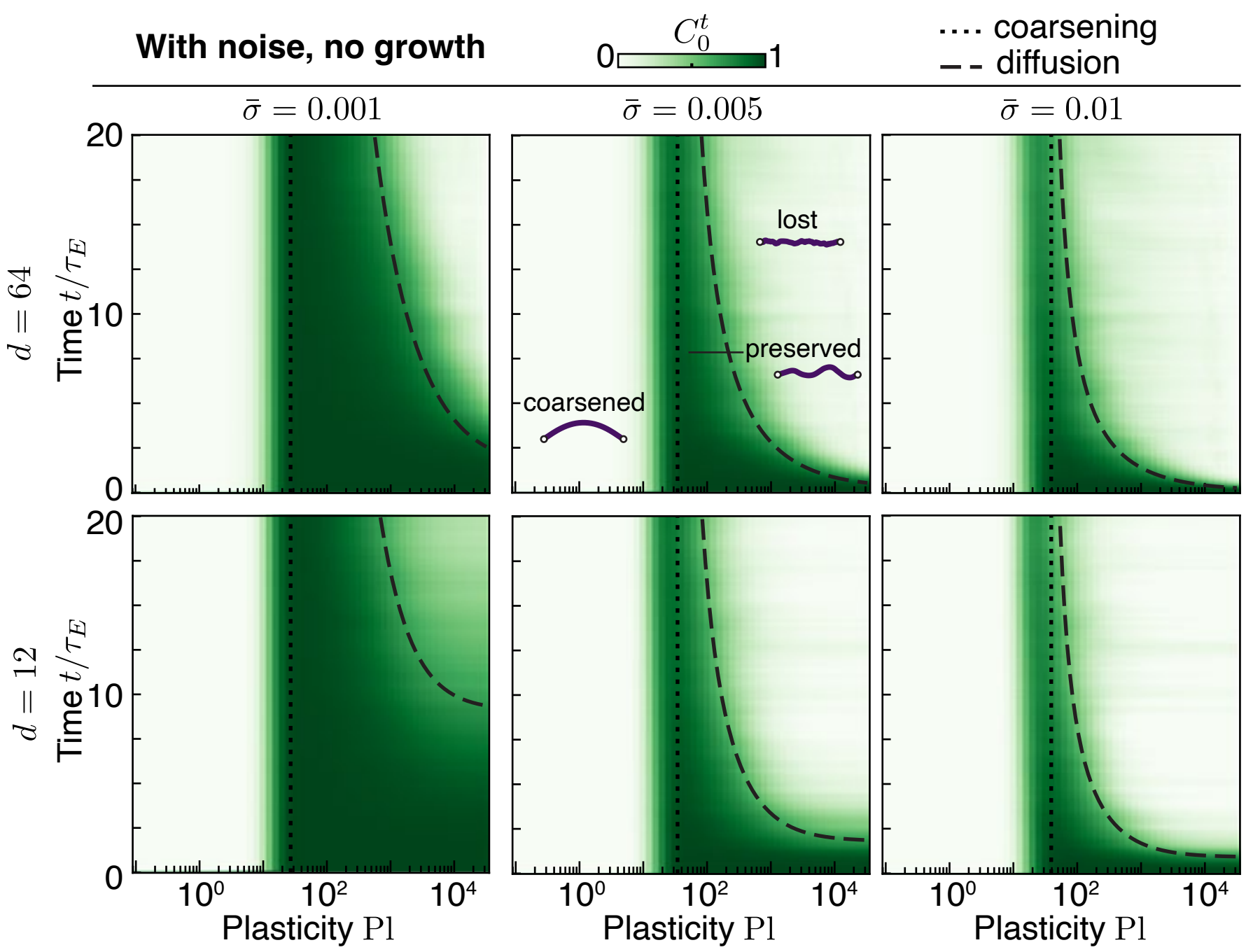

FIG. S2. Theoretical regime bounds are valid across different noise amplitudes and mode numbers in the absence of growth. Results averaged over 96 replicates; initial pattern has $m = 4$, and we keep $L/L_0 = 1.1$. We vary $d = 64$ (top row) and $d = 12$ (bottom row), and noise amplitude $\bar{\sigma} \in [0.001, 0.005, 0.01]$ from left to right column. Small-angle formulation.

the integral

$$\phi_n(t) = \phi_n(0)e^{-\eta t} + \int_0^t \frac{\mathrm{d}\tau}{\eta} e^{-\eta(t-\tau)}\theta_n(\tau). \tag{S29}$$

When η is large we can ignore the initial condition as $\eta t \gg 1$ and Taylor-expand using Watson's lemma

$$\phi_n(t) = \int_0^t \frac{\mathrm{d}\tau}{\eta} e^{-\eta(t-\tau)} \left(\theta_n(t) + (\tau - t)\dot{\theta}_n(t) + O((\tau - t)^2)\right) \tag{S30}$$

$$= \theta_n(t) - \frac{1}{\eta}\dot{\theta}_n(t) + O\left(\frac{1}{\eta^2}\right) \tag{S31}$$

Inserting this relationship into Eq. (S8), we find the effective dynamics

$$\gamma_n \dot{\theta}_n = F\theta_n + \sqrt{\frac{2\sigma}{L}}\zeta_n(t), \text{ with } \gamma_n = \mu + \frac{Bq_n^2}{\eta}. \tag{S32}$$

To find F, we differentiate the constraint $\sum_n \theta_n^2 = C$, which ignoring the noise, leads to

$$F\left(\sum_n \frac{\theta_n^2}{\mu + Bq_n^2/\eta}\right) = 0 \tag{S33}$$

which implies $F = 0$ at lowest order in $1/\eta$ and noise since the sum is positive. Thus, in the absence of growth, the large-plasticity dynamics have no deterministic drift at leading order: once the rest shape tracks the current shape, every point on the constraint sphere $\sum_n \theta_n^2 = C$ is marginally stable. When noise is present, the constraint projects the stochastic fluctuations onto this sphere, so the modal state undergoes the anisotropic Brownian motion described in the main text. The anisotropy is set by the mode-dependent mobility γ_n^{-1}: high-wavenumber modes have larger γ_n and therefore diffuse more slowly, whereas $\gamma_n \to \mu$ for all n as $\eta \to \infty$, yielding isotropic diffusion. This noise-driven motion away from the initial modal state is the mechanism responsible for the loss of "shape memory" at high plasticity. Its characteristic timescale $t_D \sim D_d^{-1}$ sets the high-plasticity diffusion boundary shown in Fig. 2c of the main text and Fig. S2; the dependence of t_D on the total number of modes d and noise amplitude σ is consistent with numerical observations (Fig. S2).

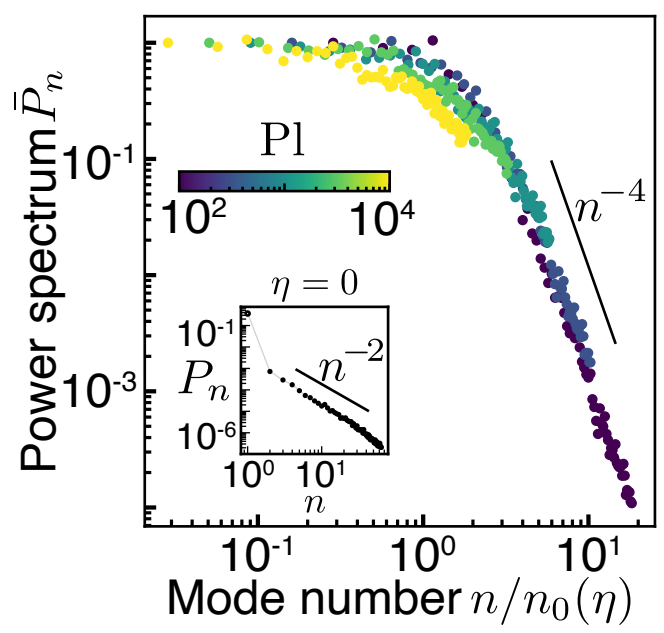

FIG. S3. In the presence of noise, plastic systems converge to a late time power spectrum $\bar{P}_n = \langle\theta_n^2\rangle/\langle\theta_1^2\rangle$, with higher plasticity leading to uniform spectral distributions. Inset shows the late-time spectrum for elastic dynamics $\eta = 0$. Here, $d = 64$, $L/L_0 = 1.1$, and $\bar{\sigma} = 0.005$.

C. Steady-state spectrum

At long times $t/\tau_E \to \infty$, anisotropic diffusion on the constraint sphere leads to an inhomogeneous probability distribution. To characterize the statistical steady state, we consider the power spectrum $\langle\theta_n^2\rangle$, which reads

$$\langle\theta_n^2\rangle = C\frac{1/\gamma_n^2}{\sum_k 1/\gamma_k^2} \propto \frac{1}{\left(1+(n/n_0)^2\right)^2}. \tag{S34}$$

with a crossover mode number $n_0 = \sqrt{\eta\mu L^2/(\pi^2 B)} = (L/L_0)\sqrt{\mathrm{Pl}}/\pi$: slow-diffusing high-frequency modes are less weighted. This spectrum tends to become flatter as $\eta \to \infty$ and the diffusion becoming homogeneous, with a sharper drop-off with n than in the elastic regime $\mathrm{Pl} \ll 1$: In the elastic case, we recover the standard result for elastic beams under tension that noise generates a power spectrum peaked at $n = 1$, with higher modes decaying as $\langle\theta_n^2\rangle = D/(n^2-1) \sim 1/n^2$ for $n > 1$ compared to n^{-4} at intermediate plasticity [1]. These results are consistent with the observations of Fig. S3. Note that the curves do not perfectly collapse onto each other: this is due to the tension F not vanishing exactly at lower Pl in the presence of noise.

III. DERIVATION OF MEMORY CRITERION FOR THE GROWING PLASTICA

We consider a model where growth changes the length of the rod as $L(t) = L_1 e^{gt}$, with g the growth rate, while giving a constant bending rigidity B. In this case, growth generates a baseline tension $T = \mu\dot{C}/(2C) \propto g$, but also leads the elastic coarsening rate $Bq_n^2/\mu \sim 1/L(t)^2$ and spectral noise amplitude $\sqrt{2\sigma/L(t)}$ to decay over the growth period. In what follows, we derive explicit expression for the recall criteria as a function of the plastic rate η and geometric parameters.

A. Elastic regime

First, we consider the deterministic criterion for the low plasticity regime. The dynamics for the n-th mode n when the m-th mode is dominant are now given by

$$\mu\dot{\theta}_n = \left(-Bq_n^2(t) + \frac{\mu\dot{C}}{2C} + Bq_m^2(t)\right)e^{-\eta t}\theta_n \tag{S35}$$

where now $q_n(t) = \pi n/L(t)$ decreases with time. The mode fractions $r_n = \theta_n^2/C(t)$ then evolve under

$$\dot{r}_n = 2\frac{\theta_n\dot{\theta}_n}{C} - \frac{\dot{C}}{C}\frac{\theta_n^2}{C} \tag{S36}$$

$$= \left(-\frac{2Bq_n^2}{\mu}(t) + \frac{2Bq_m^2(t)}{\mu}\right)e^{-\eta t}r_n. \tag{S37}$$

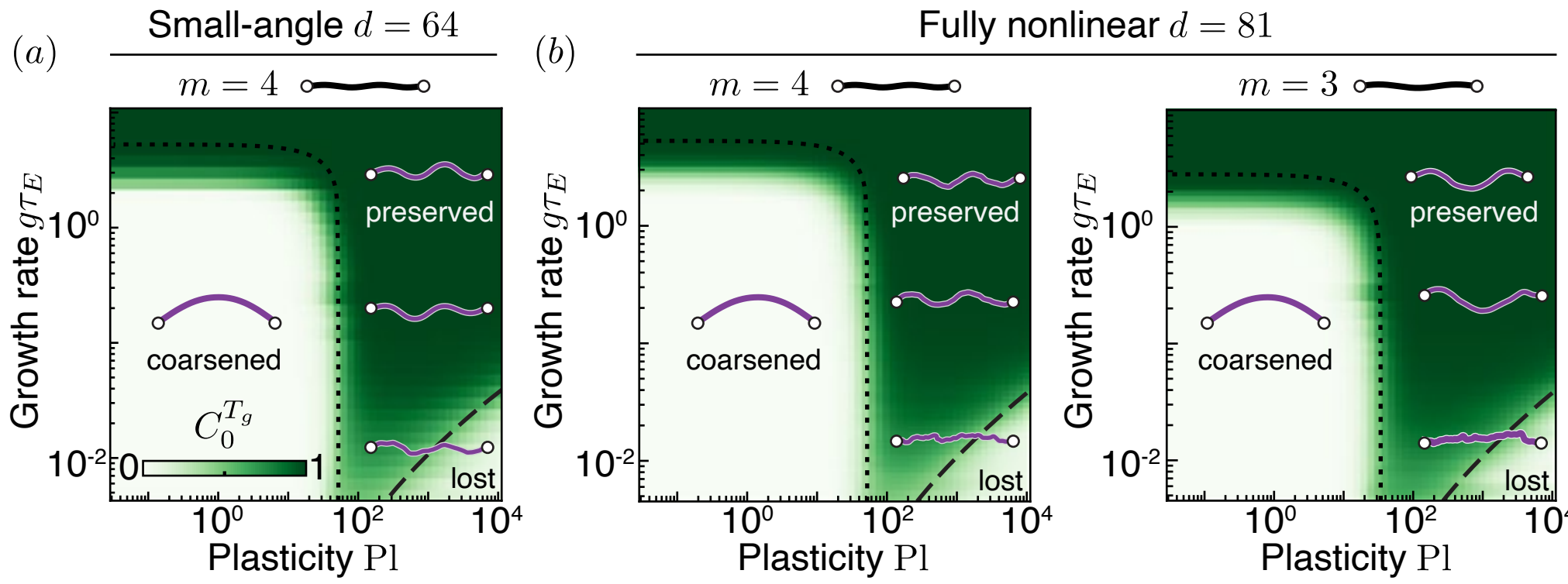

FIG. S4. Fully-nonlinear and small-angle simulations both agree with theory in the presence of growth. (a) Small-angle simulations with increased mode number $d = 64$ compared to main text Fig. 3. All other parameters identical to Fig. 3a: $L_0 = 1$, $L_1 = 1.005$, $L_f/L_1 = 1.1$, $\bar{\sigma} = 0.001$ and averaged over 96 simulations . (b) Fully-nonlinear simulations give similar phenomenology and agreement with theory. $L_0 = 1$, $L_1 = 1.005$, $L_f/L_1 = 1.1$, $\bar{\sigma} = 0.001$, results are averaged over 48 simulations.

The main difference with the no-growth case is the decaying effective bending coefficient as $L(t)$ increases, which adds to the η-driven vanishing of the growth rate. The total amplification for a finite growth time $T_g = \ln(L_f/L_1)/g$ is then given by

$$\ln \frac{r_n(T_g)}{r_n(0)} \geq \int_0^{T_g} \mathrm{d}t \frac{2B\pi^2}{\mu L_1^2} e^{-(2g+\eta)t}(m^2 - n^2) = \frac{2B\pi^2}{\mu L_1^2} \frac{m^2 - n^2}{2g + \eta}(1 - e^{-(2g+\eta)T_g}) \tag{S38}$$

$$= \frac{2B\pi^2}{\mu L_1^2} \frac{m^2 - n^2}{2g + \eta} \left[1 - \left(\frac{L_1}{L_f}\right)^{2+\eta/g}\right] \tag{S39}$$

To have $r_1(T_g) \leq 1$ for $r_1(0) = \epsilon^2 < 1$ then requires the condition

$$\frac{2B\pi^2}{\mu L_1^2} \frac{m^2 - n^2}{2g + \eta} \left[1 - \left(\frac{L_1}{L_f}\right)^{2+\eta/g}\right] \leq \ln(1/\epsilon^2) \tag{S40}$$

Even when $\eta = 0$, there exists a growth rate g_0 such that coarsening is interrupted – it is well known that rapid compression can stabilize higher order modes – with this critical growth rate given by

$$g_0 = \frac{B\pi^2}{\mu L_1^2 \ln(1/\epsilon^2)}(m^2 - 1)\left(1 - \frac{L_1^2}{L_f^2}\right). \tag{S41}$$

We find that both the fully nonlinear and small-angle simulations agree with the theory in the presence of growth (Fig. S4).

B. Large-plasticity regime with growth

The noise-driven effect persists even with growth tension, after rescaling by $C(t)$. In the presence of noise, in the large plasticity regime we still have

$$\gamma_n \dot{\theta}_n = F\theta_n + \sqrt{\frac{2\sigma}{L}}\zeta_n(t) \tag{S42}$$

Satisfying $\sum_n \theta_n^2 = C(t)$ now requires

$$\dot{C} = 2F \sum_n \frac{\theta_n^2}{\gamma_n} = 2FC \sum_n \frac{r_n}{\gamma_n} \tag{S43}$$

with $r_n = \theta_n^2/C(t)$ the mode fraction, such that $\sum_n r_n = 1$ at all times. Solving for the tension gives a result proportional to the mode occupancy averaged mobility

$$F = \frac{\dot{C}}{2C}\frac{1}{\langle\gamma^{-1}\rangle}, \quad \langle\gamma^{-1}\rangle = \sum_n \frac{r_n}{\gamma_n} \tag{S44}$$

We now investigate the deterministic and stochastic dynamics engendered by this effect.

1. *Growth in the large-plasticity regime produces limited coarsening*

The growth-induced nonzero tension leads the deterministic dynamics to obey a replicator equation with fitness $1/\gamma_n$,

$$\dot{r}_n = \frac{2}{C}\theta_n\dot{\theta}_n - \theta_n^2\frac{\dot{C}}{C^2} \tag{S45}$$

$$= \frac{\dot{C}}{C}\left(\frac{1/\gamma_n}{\langle\gamma^{-1}\rangle} - 1\right) r_n \tag{S46}$$

driving the mode fraction to be dominated by slow growing modes. This growth-induced tension thus leads to a similar deterministic coarsening effect as in the elastic limit, but how important is this contribution to coarsening? Near mode-m dominance, $\langle\gamma^{-1}\rangle \approx 1/\gamma_m$, the fundamental mode fraction grows as

$$\frac{\dot{r}_1}{r_1} \approx \frac{\dot{C}}{C}\left(\frac{\gamma_m}{\gamma_1} - 1\right) \tag{S47}$$

The cumulative coarsening due to this deterministic drift is given by

$$\ln(r_1(T_g)/r_1(0)) \leq \int_0^{T_g} \mathrm{d}t\, \frac{\dot{C}}{C} e^{-2gt}\frac{B\pi^2(m^2-1)}{\mu\eta L_1^2}$$

$$= \int_0^{T_g} \mathrm{d}t\, \frac{gL_0e^{-2gt}}{L_1e^{gt} - L_0}\frac{B\pi^2(m^2-1)}{\mu\eta L_1^2} \approx \frac{B\pi^2(m^2-1)}{\mu\eta L_0^2}\left[-\ln\left(1 - \frac{L_0}{L_1}\right) - \left(\frac{L_0}{L_1}\right) - \frac{1}{2}\left(\frac{L_0}{L_1}\right)^2\right] \tag{S48}$$

with the last approximation holding when integrated to infinity. Interestingly, this last result does not depend on the growth rate g nor on the final length. Up to a geometry-dependent factor, this is the same coarsening criterion as in the elastic regime. For $L_1/L_0 \gg 1$, we have to leading cubic order

$$\mathrm{Pl}_c^\eta = \frac{\pi^2(m^2-1)}{6\ln(1/\epsilon)}\left(\frac{L_0}{L_1}\right)^3 + O\left(\left[\frac{L_0}{L_1}\right]^4\right) \approx \frac{1}{6}\mathrm{Pl}_c^{\mathrm{elastic}}\left(\frac{L_0}{L_1}\right) \to 0 \tag{S49}$$

while for $L_1/L_0 = 1.005$ as in our simulations $\mathrm{Pl}_c^\eta \approx 1.9\mathrm{Pl}_c^{\mathrm{elastic}}$. This effect is strongest at small η where the validity of the high plasticity approximation is less clear, and it corrects the lower bound on η to prevent elastic coarsening. Hence, at high η noise-driven mode mixing is still dominant, and this coarsening effect can be safely ignored.

2. *State diffusion with growth*

The results above indicate that we can still ignore the effect of deterministic coarsening at large plasticity, which is then still dominated by the effect of stochastic mode diffusion. We now compute the timescale of diffusion with $g > 0$. In the presence of growth, the diffusion coefficient is now time-dependent as length varies. The total angular drift is now given by

$$\Phi(T_g) = \int_0^{T_g}(d_{\mathrm{eff}}(t) - 1)D_0(t)\mathrm{d}t \approx \int_0^{T_g}(d_{\mathrm{eff}}(0) - 1)D_0(t)\mathrm{d}t \tag{S50}$$

$$= \int_0^{T_g}\frac{(d_{\mathrm{eff}}(0) - 1)\sigma}{\mu^2\Delta(t)}\mathrm{d}t = \frac{(d_{\mathrm{eff}}(0) - 1)\sigma}{\mu^2 gL_0}\ln\left(\frac{(L_f - L_0)L_1}{(L_1 - L_0)L_f}\right) \tag{S51}$$

where L_1 is the length at time $t = 0$. Φ has to be ≤ 1 for preservation of the initial condition. In the large η limit, $d_{\text{eff}} \to d$ from below and there is thus a critical growth rate above which diffusion cannot happen fast enough to lose the initial condition

$$g_\infty = \frac{(d-1)\sigma}{\mu^2 L_0} \ln\left(\frac{(L_f - L_0)L_1}{(L_1 - L_0)L_f}\right). \tag{S52}$$

Simulations in the small-angle regime quantitatively agree with fully nonlinear simulations and the theory (Fig. S4).

IV. ALTERNATIVE MODEL WITH EXTERNAL VISCOSITY

Our results above and in the main text assume an internal viscosity of the material. To understand how different damping and dynamics affect the results above, in this section we study the plastica in the case where the dominant cause of dissipation is external viscosity from a surrounding medium. In the small slope limit, where the curve centerline $\mathbf{r}$ can be parametrized as a function $\mathbf{r}(x) = (x, h(x))$ and $\partial_x h = \theta \ll 1$, the overdamped case has linear momentum balance $\mathbf{F}' = (0, \mu\dot{h})^T$. We then have $F_x = F$ a constant in x and $\partial_x F_y = \mu\partial_t h$, which leads to the the dynamical equations

$$\mu\partial_t h = -B(\partial_x^4 h - \partial_x^2 \kappa_0) - F\partial_x^2 h \tag{S53a}$$

$$\partial_t \kappa_0 = \eta(\partial_x^2 h - \kappa_0) \tag{S53b}$$

subject to the inextensibility constraint

$$L = \int_0^{L_0} \mathrm{d}x\, \sqrt{1 + (\partial_x h)^2} \approx L_0 + \frac{1}{2}\int_0^{L_0} \mathrm{d}x\, (\partial_x h)^2 \tag{S54}$$

We again consider hinged (simply supported) boundary conditions such that $h = \partial_{xx}^2 h = 0$ at both ends. Expanding in sine modes with $q_n = \pi n/L_0$, we have

$$h(x,t) = \sum_{n=1}^{N} h_n(t)\sin(q_n x), \quad \kappa_0(x,t) = \sum_{n=1}^{N} -q_n^2 c_n(t)\sin(q_n x). \tag{S55}$$

In mode space, the inextensibility constraint $(1/2)\int_0^{L_0} \mathrm{d}x\,(\partial_x h)^2 = L - L_0$ becomes

$$\sum_{n=1}^{N} q_n^2 h_n^2 = 4\frac{\Delta(t)}{L_0} \tag{S56}$$

and the mode amplitudes now evolve under

$$\mu\dot{h}_n = (-Bq_n^4 + q_n^2 F)h_n + Bq_n^4 c_n, \tag{S57a}$$

$$\dot{c}_n = \eta(h_n - c_n). \tag{S57b}$$

The tension F can be found by time-differentiating the inextensibility constraint, leading to

$$\frac{1}{\mu}\sum_n q_n^2\left[h_n(-Bq_n^4 + q_n^2 F)h_n + h_n B q_n^4 c_n\right] = \frac{2\dot{\Delta}}{L_0} \tag{S58}$$

which gives

$$\left(\sum_n q_n^4 h_n^2\right) F = \mu\frac{2\dot{\Delta}}{L_0} + B\left(\sum_n q_n^6 h_n(h_n - c_n)\right) \tag{S59}$$

This tension leads to qualitatively similar dynamics, with faster coarsening and an elliptic, rather than spherical, constraint manifold. The criterion for plasticity to prevent elastic coarsening of a pure mode m, which has tension $F = Bq_m^2$ can be found by considering the new replicator for the mode $r_n = q_n^2 h_n^2/C$, now weighted by the wavenumber squared

$$\mu\dot{r}_n = 2Bq_n^2(q_m^2 - q_n^2)r_n \tag{S60}$$

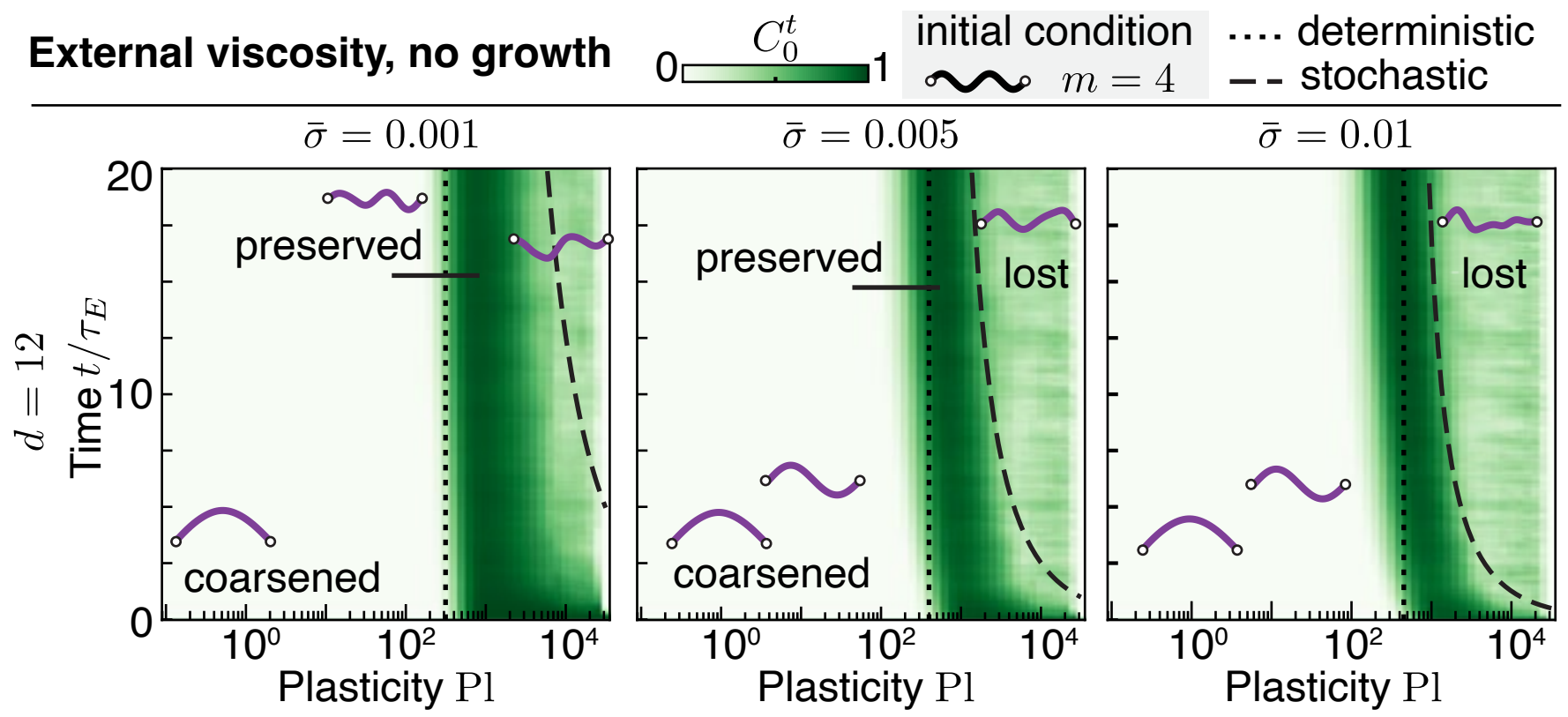

FIG. S5. Dynamics of recall for the plastica with external viscosity. We find similar phenomenology as the internal viscosity, with modified thresholds and observe intermediate coarsening for $10 \lesssim \mathrm{Pl} \lesssim 10^3$ for the larger noise levels. Here we use the Pearson correlation of $r_n = q_n^2 h_n^2$ to weight spectral components comparably to the internal viscosity case.

which leads to a modified recall criterion

$$\ln(r_1/\epsilon^2) = \int_0^\infty \frac{2B}{\mu} q_1^2 (q_m^2 - q_1^2) e^{-\eta t} \mathrm{d}t = \frac{2\pi^4 B}{\mu \eta L_0^4}(m^2 - 1) \le \ln(1/\epsilon^2). \tag{S61}$$

This criterion is valid at low noise when the dominant escape channel is towards the fundamental mode. At higher noise, the system can also visit partially coarsened state and stay frozen there, as the transition rates between modes $m \to m-1$ are faster than transitions between $m \to 1$ by a ratio $\rho = (m-1)(2m-1)/(m+1) > 1$. Further work could explore the phase diagram of memory retention in the presence of these more complex transients.

The large-plasticity stochastic dynamics now read, in the absence of growth, as

$$(\mu + Bq_n^4/\eta)\dot{h}_n = \sqrt{\frac{2\sigma}{L}} \zeta_n \tag{S62}$$

which means that the rescaled mode $u_n = n h_n$ diffuses on a constraint sphere with a modified effective diffusion

$$D_d = (d_{\text{eff}} - 1) D_0, \qquad d_{\text{eff}} = \sum_n \frac{n^2}{(1 + Bq_n^4/(\mu\eta))^2} \tag{S63}$$

with $D_0 = \sigma/(\Delta\mu^2)$. Despite the more complex transients, the resulting criteria yield satisfactory boundaries (Fig. S5).

---

[1] Masao Doi and Samuel F. Edwards. *The theory of polymer dynamics*. Number 73 in International series of monographs on physics. Clarendon Press, Oxford, reprint edition, 2013.
